# Point-of-care measurement of activated clotting time for cardiac surgery as measured by the Hemochron signature elite and the Abbott i-STAT: agreement, concordance, and clinical reliability

**DOI:** 10.1186/s12871-019-0846-z

**Published:** 2019-09-06

**Authors:** Daniel Dirkmann, Elisabeth Nagy, Martin W. Britten, Jürgen Peters

**Affiliations:** Klinik für Anästhesiologie und Intensivmedizin, Universität Duisburg-Essen & Universitätsklinikum Essen, Hufelandstraße 55, D-45122 Essen, Germany

**Keywords:** Activated clotting time, ACT, Method comparison, Anticoagulation, Cardiopulmonary bypass, Heparin, Protamine

## Abstract

**Background:**

Since inadequate heparin anticoagulation and insufficient reversal can result in complications during cardiopulmonary bypass (CPB) surgery, heparin anticoagulation monitoring by point-of-care (POC) activated clotting time (ACT) measurements is essential for CPB initiation, maintainance, and anticoagulant reversal. However, concerns exist regarding reproducibility of ACT assays and comparability of devices.

**Methods:**

We evaluated the agreement of ACT assays using four parallel measurements performed on two commonly used devices each (i.e., two Hemochron Signature Elite (Hemochron) and two Abbott i-STAT (i-STAT) devices, respectively). Blood samples from 30 patients undergoing cardiac surgery on CPB were assayed at specified steps (baseline, after heparin administration, after protamine administration) with four parallel measurements (two of each device type) using commercial Kaolin activated assays provided by the respective manufactures. Measurements were compared between identical and different device types using linear regression, Bland-Altman analyses, and calculation of Cohen’s kappa coefficient.

**Results:**

Parallel i-STAT ACTs demonstrated a good linear correlation (r = 0.985). Bias, as determined by Bland-Altman analysis, was low (− 3.8 s; 95% limits of agreement (LOA): − 77.8 -70.2 s), and Cohen’s Kappa demonstrated good agreement (kappa = 0.809). Hemochron derived ACTs demonstrated worse linear correlation (r = 0.782), larger bias with considerably broader LOA (− 13.14 s; 95%LOA:-316.3–290 s), and lesser concordance between parallel assays (kappa = 0.554). Although demonstrating a fair linear correlation (r = 0.815), parallel measurements on different ACT-devices showed large bias (−20s; 95% LOA: − 290-250 s) and little concordance (kappa = 0.368). Overall, disconcordant results according to clinically predefined target values were more frequent with the Hemochron than i-STAT. Furthermore, while discrepancies in ACT between two parallel iSTAT assays showed little or no clinical relevance, deviations from parallel Hemochron assays and iSTAT versus Hemochron measurements revealed marked and sometimes clinically critical deviations.

**Conclusion:**

Currently used ACT point-of-care devices cannot be used interchangeably. Furthermore, our data question the reliability of the Hemochron in assessing adequacy of heparin anticoagulation monitoring for CPB.

## Background

Unfractionated Heparin (UFH) is the standard anticoagulant for cardiac surgery involving cardiopulmonary bypass (CPB) circuits since 1953 [1]. Up to the 1970s whole blood clotting time was used to monitor Heparin’s anticoagulant effects. Ever since, activated partial thromboplastin time and anti-factor-Xa assays are considered the gold standard for monitoring Heparin induced anticoagulation [2]. However, long turnaround times [3] and the high heparin concentrations required to prevent blood clotting in the bypass circuit hamper the intraoperative usefulness of these latter assays in cardiac surgery. Following its initial description by Hattersley in 1966 [4], the Activated Coagulation Time (ACT) has been developed to be usable as a point of care (POC) test. Most devices, like the Hemochron Signature Elite (Accriva, San Diego, CA, USA) used in our study, assess the ACT by mechanical or photo-optical detection of thrombin induced clot formation. Another method, as used in the i-STAT (Abbot Point-of-Care, Princeton, NJ, USA), is based on the electrochemical detection of a product derived from thrombin dependent cleavage of a substrate other than fibrinogen (Phenylalanin-Pipecolyl-Arginin-NH_3_-C_6_H_4_-NH-C_6_H_4_-OCH_3_). [5] While the former methods are affected by thrombin generation, platelet aggregation, and fibrin polymerization, the latter assay only depends on thrombin generation, potentially resulting in measurements more precisely reflecting Heparins anti-thrombin properties. Although both methods are approved for clinical use, their reproducibility, agreement, and interchangeability have not been well investigated so far.

## Methods

To assess the agreement, concordance, and interchangebility of ACT measurements for cardiac surgery two commonly used devices for ACT monitoring were compared assessing deviations between measurements on the same device as well as between different devices, i.e., an i-STAT device was compared to the Hemochron. Ethics committee approval (Ethik-Kommission Medizinische Fakultät der Universität Duisburg-Essen, Robert-Koch-Straße 9–11, D-45147 Essen, Germany, protocol no. 16–7027-BO) was obtained and informed consent of patients was waived because of pseudomisation of all data.

### ACT measurements

ACT measurements were performed in parallel in the same sample at specified intraoperative time points, i.e., prior to and after heparin administration for CPB, and after CPB following protamine infusion. According to institutional standards ACT was assessed once prior to heparin administration and at least once following protamine infusion. Additional measurements following protamine infusion were performed as considered necessary by the anesthesiologist in charge. After heparin administration, i.e., during CPB, ACT was assessed at least every 30 min. Further analyses were performed 5 min after any additional heparin had been given or as deemed necessary. Arterial blood was drawn from an arterial line or from the oxygenated blood returning line from the CPB circuit while on bypass. After discarding the first 5 mL, 2 mL of blood were drawn into a standard, unheparinized syringe. Afterwards, ACT measurements were run immediately and in parallel on two prepared i-STAT and two Hemochron devices in random order, according to the anesthesiologist in charge.

According to the manufacturer, the ACT measurement range is 0–1005 s for the Hemochron and 50–1000 s for the i-STAT, respectively.

### Heparin / protamine management

Heparin (400 U per kg body weight) was administered intravenously so to achieve an ACT greater 400 s before clearance to commence CPB. The pump-prime contained an additional 5000 U of Heparin and additional injections of 50–100 U heparin per kg body weight were given as required to keep the ACT above 430 s during CPB. If the target ACT was not obtained despite repeated heparin injections, 500–1000 IU of antithrombin were injected. After successful weaning form CPB protamine (300 U per kg body weight) were infused for heparin antagonization.

### Data analyses

Data were analyzed comparing the ACT results of identical devices, i.e., comparing a) the results of the two Hemochron devices; b) the results of the two i-STAT devices, respectively, and also c) the results of the two different device, i.e., i-STATs and Hemochron devices. For the latter comparison, data obtained using each of the Hemochron devices (designated as Hemochron_1_ and Hemochron_2_, respectively) were compared to both corresponding data points obtained using either i-STAT device (designated as i-STAT_1_ and i-STAT_2_, respectively).

Data were analyzed by fitting linear regressions and calculating Spearman’s correlation coefficient rho. Furthermore, Bland-Altman analyses were performed to calculate the mean difference (bias) ± 95 limits of agreement (LOA) between the results of the respective assays. Calculation of Cohen’s kappa was performed to assess the concordance of the two respective devices in rating the ACT as above or below a predefined clinical cut-off. Therefore, measurements were considered concordant if results yielded by both devices were either uniformly above or below 430 s during CPB and above or below 120 s prior to heparin injection as well as post protamine infusion, respectively. Furthermore, results were also considered concordant, if both devices consistently indicated an ACT above the respective measuring range. As suggested by Altman, concordance was considered “insufficient” for a Kappa ≤0.2, “sufficient” with a Kappa of 0.21–0.4, “fair” with a Kappa of 0.41–0.6, “good” with a Kappa of 0.61–0.8, and “very good” with a Kappa of 0.81–1.0, respectively [6].

## Results

Data were obtained from 30 patients undergoing various cardiac surgeries using cardio-pulmonary bypass (Table 1). Data were available for 190 meassurement points, with 163 data sets including complete data for all four parallel assay. In 27 data sets, data from one or two ACT devices were missing. Finally, comparisons were made based on 181 parallel data sets for the i-STAT and 167 results for the Hemochron device, respectively. For the comparison of i-STAT and Hemochron data a total of 690 pairs of values were available.

**Table 1:** Demographics of the study population

Gender (number; male/female)	19 / 11
Age (years; mean ± SD)	70,5 ± 11
Height (cm; mean ± SD)	170,2 ± 8,8
Weight (kg; mean ± SD)	80,3 ± 17,3
Type of surgery	(n)
Coronary artery bypass surgery alone	13
Coronary artery bypass + aortic valve surgery	5
Coronary artery bypass + mitral valve surgery	1
Coronary artery bypass + ascending aortic surgery	1
Aortic valve surgery	3
Aortic valve + ascending aortic surgery	4
Mitral valve surgery	3

Data are numbers or means ± standard deviation

### ACT measurements with the i-STAT device

**Fig. 1:**
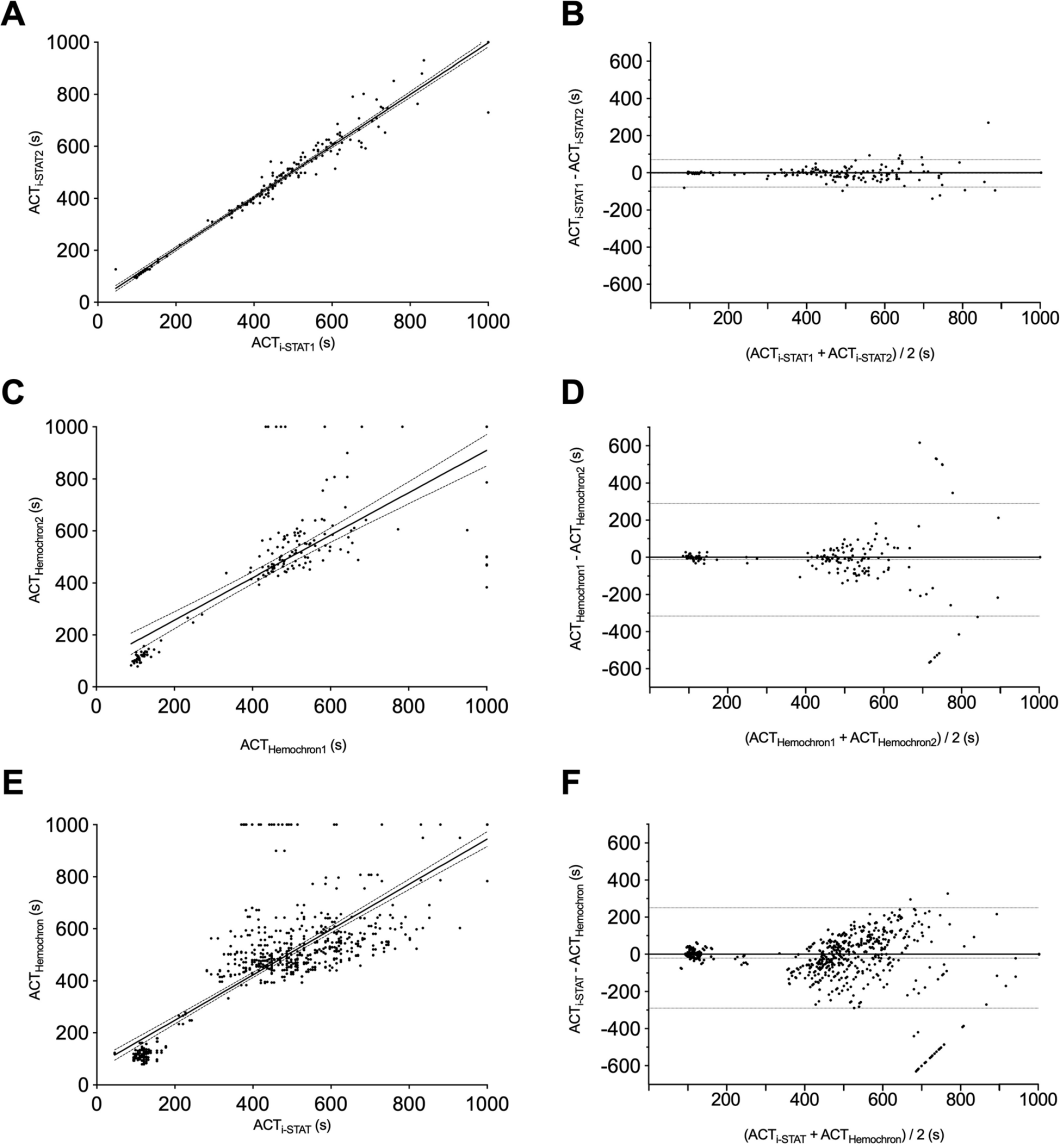
Linear correlation and Bland-Altman-Plots for comparison of results from the two i-STAT devices (pannel **a** and **b**), the two Haemochron signature elite devices (panels **c** and **d**), and for results from i-STATs and Haemochron assays (pannel **e** and **f**)

**Fig. 2:**
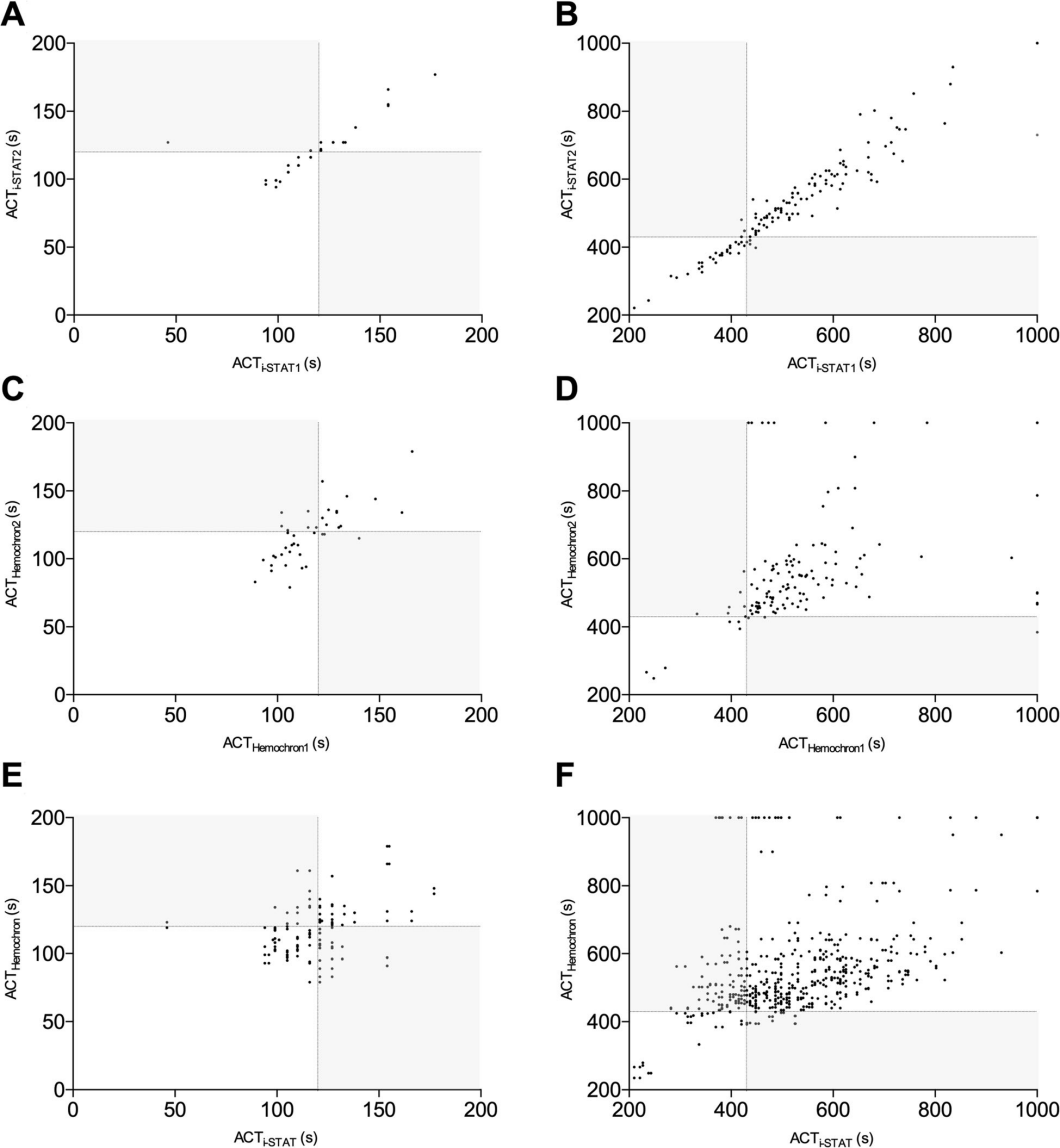
Linear correlation for the ACT measurement below 200 s (panels **a**, **c,** and **e**), for the whole measurement range up to 1000 s (panels **b**, **d**, and **f**), for comparison of the ACT measurements from the two i-STAT devices, the two Haemochron devices, and for the results from i-STAT and Haemochron assays respectively. Dotted lines indicate the respective clinical target values (i.e.,120 s; pannels **a**, **c**, and **e**; and 430 s; panels **b**, **d**, and **f**), respectively. Disconcordant, clinically relevant discrepant resulsts are depicted in the light grey fields

Comparison of parallel results obtained with the two i-STAT devices revealed a very good linear correlation (r = 0.985) (Fig. 1a) and Bland-Altman analyses demonstrated good agreement (bias: − 3.796 s; 95%-confidence interval: − 77.80s – 70.21 s) (Fig. 1b). Overall, 91.16% results were considered concordant according to the clinically relevant predefined criteria described above. Cohen’s Kappa coefficient for the agreement between both i-STAT devices was 0.809 for all measurements, 0.836 for results below 200 s, and 0.776 for ACT measurements above 200 s, respectively, demonstrating a good to very good concordance. Not-matching results in parallel i-STAT assays were found in 6.3% (3 of 48) (mean difference: 5 s) of ACTs less than 200 s and in 6.8% (9 out of 132) in ACTs greater 200 s (mean difference: 23.3 s; range: 11–61 s) (Fig. 2). An ACT of ≥1000 s was recorded three times. While on two of these occasions results on both devices were concordant in exceeding 1000 s, the parallel results in the third case were ≥ 1000 s and 730 s.

### ACT measurements with the Hemochron signature elite

Comparison of data obtained using in parallel two Hemochron devices revealed a linear correlation (r = 0.78) (Fig. 1c). Bland-Altman analyses demonstrated good agreement, albeit with a relatively broad 95%-confidence interval (bias: − 12.14 s; 95%-confidence interval: 316.3–290 s) (Fig. 1d). 81.33% of the results were considered concordant and Cohens’s Kappa coefficient demonstrated moderate concordance for all measurements (Kappa = 0.554). Concordance for the ACT values less than 200 s was similar (Kappa: 0.553) while concordance for the ACTs above 200 s was sufficient (Kappa 0.347). 21.4% (9 of 42) of ACTs < 200 s (mean difference: 13.8 s; range: 4–32 s) and 17.6% (22 out of 125) of ACTs> 200 s (mean difference: 57.9 s; range: 2–138 s) were incongruent (Fig. 2). Of note, ACT values greater than 1000 s were recorded on one of the Hemochron devices in 16 parallel assays. Two of these measurements were concordantly assessed as exceeding 1000 s also on the other device. The mean difference with the in parallel assay run was 468 s (213 s and 616 s). Of note, in one case the parallel result was only 384 s.

### Comparison of i-STAT and Hemochron data

Linear correlation between these two different devices demonstrated a slightly better correlation (r = 0.815) (Fig. 1e) than comparison of two Hemochron devices, but a larger bias (− 20 s; 95%-confidence interval: − 290–250 s) (Fig. 1f). 71.2% of all results were considered concordant. Concordance, as calculated from Cohen’s Kappa coefficient was poor (Kappa = 0.368). For ACTs values less than 200 s as much as 28.8% (51 of 177) and for ACTs exceeding 200 s as much as 26.5% (136 out of 514) of the results were considered inconsistent. ACT values ≥1000 s were recorded in 36 readings (Fig. 2). While results were rated concordant by the other device in 10 of these readings, the difference between the i-STAT and the Hemochron device values ranged between 120 and 630 s in the other 26 readings. In 10 measurements using the i-STAT ACT was less than 430 s and in 7 of these cases even less than 400 s (Fig. 2).

## Discussion

Fast, precise, and reliable assessment of sufficient Heparin evoked anticoagulation and its reversal by Protamine are essential for safely conducting surgical procedures requiring cardiopulmonary bypass. Insufficient anticoagulation can result in life-threatening events like clot formation within the bypass circuit or oxygenator and other thrombotic or thromboembolic complications [7]. Furthermore, residual heparin following Protamine administration and a Protamine overdose may be associated with increased bleeding [8, 9].

Although the ACT is considered the clinical standard for intraoperative POC monitoring of heparin-evoked anticoagulation, wide variability exists regarding institutional target values considered safe to conduct CPB, usually ranging from 300 to 600 s [10]. This broad range in target ACTs is, at least in part, considered to have resulted from the use of different measurement devices and also of various assays, using either Celite or Kaolin as the contact activator of coagulation [11]. In fact, ACT assays have not been standardized yet and probably never will, hampering comparability.

Data on the reproducibility of ACT tests using simultaneous measurements on identical device types and comparison of results between different device types are sparse. Most studies were conducted using older ACT devices [7, 12], were limited to older, Celite-activated assays, [13] or compared Celite and Kaolin activated assays. [14] In addition, prior studies included patients undergoing cardiac catheterization [5, 15, 16] with lower target ACTs or used normal control plasma instead of the patients’ blood. [17] Accordingly, our study was designed to include real-life data only from patients undergoing cardiac surgery where errors in ACT POC measurements can evoke grave sequelae. Furthermore, we decided to use two of the most recent versions of the respective devices and only Kaolin activated assays.

A major issue encountered was that the results obtained when using different analyzers, albeit tending into the same direction following Heparin injection or after Protamine infusion, frequently provided markedly different results. Of note, some of these results obtained from the different devices would have affected in an important way decision making, such as clearance for CPB to commence or to administer or not additional Heparin. This issue has also been addressed in a study [5] comparing single but in parallel measurements using the Kaolin activated i-STAT and the Medtronic ACT plus assays in 121 simultaneously drawn blood samples (59 from the cardiac catheterization laboratory and 62 during surgery, respectively). In this latter study, the authors demonstrated an high correlation (r = 0.94) between these two devices and only minor inconsistencies with respect to predefined target values. Specifically, only 2 measurements were considered incongruent but had had no impact on clinical decision making. In contrast, our study by comparing duplicate measurements on both types of analyzers, unmasked marked differences between both identical and different device types. While differences between results of parallel i-STAT-assays were of no clinical importance, a critical deviation of results was often found in parallel Hemochron assays. Specifically, one analyzer had provided an ACT of ≥1005 s while the other had already stopped at an ACT reading of 384 s. Obviously, while the former reading suggests a more than sufficient degree of heparin anticoagulation, the latter clearly is far below our target value of 430 s for CPB and, if true, would have put the patient at risk for clot formation within the CPB circuit. In fact, it is not far fetched that some of the reported CPB cases of intracardiac and oxygenator thrombosis ^18–21^ may be due to wrong ACT measurements.

The worst concordance was found for parallel i-STAT- and Hemochron assays. Here, disconcordant results were found in 28 parallel measurements with a difference between 120 and as much as 630 s. While the ACT was ≥10,005 s with the Hemochron, the parallel ACT measured with the iSTAT was below 430 s in 7 cases and even below 400 s in 5 cases. While it is impossible to recognize which assay reflected the true anticoagulant status, the lower results found with the i-STAT, if true, would have put the patient at serious risk for a potentially life threatening event while the Hemochron analysis had suggested adequate anticoagulation on several occassions. In general, it seems that the i-STAT device tends to yield lower ACT values and thus its use might result in higher heparin dosages to keep ACT values above the lower target values. However, this was not investigated in our study.

Observations regarding the comparability of another Hemochron device and the i-STAT have been made in another study conducted during the same time period as ours. This latter study contrasted ACT values obtained with the Hemochron junior in a Kaolin-activated assay with the i-STAT Celite ACT cartridge [14]. Although Celite and Kaolin-activated assays are difficult to compare, [17, 18] these authors made a similar observation with respect to the discrepancy of values, reading an ACT exceeding the upper measuring range on one device while the other read an ACT less than 1000 s. These discordant were more frequent (n = 86) with two parallel Hemochron junior devices than with two parallel i-STAT assays (n = 53). In agreement with our observations, differences between two parallel i-STAT ACT ranged between 2 and 438 s and thus can be considered of lesser clinical relevance, since all meassurements were in the safe range for CPB. In contrast, differences in parallel Hemochron Junior assays ranged between 3 and 727 s underlining the safety concerns raised by our study. Unfortunately, these authors do not provide the absolute incidence of results below a safe target value, but from their published data we can identify at least 3 such events. Accordingly, the incidence of events where one Hemochron analyzer suggests an ACT > 1005 s while a parallel assay on another device of the same type suggests inadequate anticoagulation (i.e., below 400 s), found both with the Hemochron Junior in the latter study and with the Hemochron Signature Elite in our study is alarming and questions the practice of commencing cardiopulmonary-bypass as soon as an ACT value has exceeded 400 s and the reading is ‘still running’. Rather, ACT values greater than 1005 s with any Hemochron analyzer should trigger prompt re-testing and caution in commencing CPB instead of feeling safe. The relatively great number of ACT values ≥1000 s on the i-STAT device in the latter study, [14] as compared to our observations, might be explained since in Celite activated assays the coefficient of variation increases with higher heparin concentrations potentially resulting in more ACT values exceeding the upper measurement range [17]. However, since parallel testing on another i-STAT device provided adequate less divergent results, and always within the safe range for CPB, available data suggest that ACT values ≥1000 s obtained with an i-STAT device can be considered safe.

Although with both devices there were discrepancies in the ACT values performed prior to heparin injection and post protamine infusion, these differences are of lesser clinical importance. Nevertheless, clinicians should take into account these potential differences prior to deciding on giving additional Protamine since Protamine is an antagonist against of unfractionated heparins but possesses various anticoagulatory and anti-platelet properties [13, 14]. Despite these well known effects of protamine, several institutions still use a heparin-to-protamine antagonization scheme of 1:1, although current guidelines [10] and studies [8, 19] suggest a ratio of less than 1:1 to minimize bleeding.

One potential reason for the better overall performance of the i-STAT device might be its measurement technique. The i-STAT cartridge contains a defined amount of a thrombin-specific substrate which is cleaved by the thrombin generated following activation of coagulation by kaolin. One of the cleavage products is positively charged and can be detected amperometrically. Accordingly, the ACT value from this assay likely depends mainly on thrombin generation and reflects the bloods overall thrombin generation capacity and thus also the presence or absence of anticoagulants directly or indirectly impacting on thrombin generation. In contrast, ACT measurements by Hemochron assays are based on optical detection of movement of a defined amount of coagulating blood. Accordingly, in addition to thrombin generation, measurements may also depend on the mechanical properties of the forming clot, which might be affected by various factors such as fibrinogen concentration, platelet count, hematocrit, and the presence or absence of colloids in the patients’ blood.

One might speculate whether difficulties or peculiarities during sample handling might have impacted on ACT measurement depending on the device type. However, both the Hemochron and the i-STAT device are very easy to use, were handled by well trained staff only, with no handling problems encountered. Furthermore, both devices have build-in mechanisms to detect over and underfilling. Accordingly, effects on measurements by sample volume or handling can be excluded.

## Conclusions

The i-STAT Kaolin ACT assay demonstrated better concordance in parallelly run assays than the Hemochron signature elite. Furthermore, ACT values exceeding the upper measurement range were much more frequent with the Hemochron and assays performed in parallel on identical device types demonstrated substantial bias demonstrating and an inadequate and potentially dangerous lack in reproducibility. Furthermore, concordance of the Hemochron devices with respect to clinical cut-off values is rather poor and comparison of values from different device-types, measuring the same variable, is difficult. Thus, Kaolin based ACT results obtained using the Hemochron Signature Elite and the Abbo-STAT are not interchangable and should not be used in the same institution simultaneously.

## Data Availability

Data are available from the corresponding author on request.

## Abbreviations

ACT: Activated coagulation time
CPB: Cardiopulmonary bypss
LOA: Limits of agreement
s: Seconds
